# Mass cytometry: exploring the immune landscape of systemic autoimmune and inflammatory diseases in the past fourteen years

**DOI:** 10.3389/fimmu.2024.1509782

**Published:** 2025-01-17

**Authors:** Aïcha Kante, Mathieu F. Chevalier, Damien Sène, Jeanne Chauffier, Stéphane Mouly, Benjamin Glenn Chousterman, Fériel Azibani, Benjamin Terrier, Théo Pezel, Cloé Comarmond

**Affiliations:** ^1^ Department of Internal Medicine, Lariboisière Hospital, Assistance Publique - Hôpitaux de Paris (AP-HP), Université Paris Cité, Paris, France; ^2^ INSERM UMR-S 976, Institut de Recherche Saint-Louis, Université Paris Cité, Paris, France; ^3^ INSERM UMR-S 1144, Université Paris Cité, Paris, France; ^4^ Department of Anesthesiology and Intensive Care, Lariboisière Hospital, Assistance Publique - Hôpitaux de Paris (AP-HP), Paris, France; ^5^ INSERM UMR-S 942 MASCOT - Université Paris Cité, Paris, France; ^6^ Department of Internal Medicine, Cochin Hospital, Assistance Publique - Hôpitaux de Paris (AP-HP), Université Paris Cité, Paris, France; ^7^ INSERM, U970, PARCC, Université de Paris Cité, Paris, France; ^8^ Department of Cardiology, Lariboisière Hospital, Université Paris Cité, Paris, France

**Keywords:** mass cytometry, CyTOF, imaging mass cytometry, systemic autoimmune diseases, systemic inflammatory diseases, single-cell, scRNA seq

## Abstract

Auto-immune and inflammatory diseases are heterogenous in their clinical manifestations and prognosis, even among individuals presenting with the same pathology. Understanding the immunological alterations involved in their pathogenesis provides valuable insights in different clinical phenotypes and treatment responses. Immunophenotyping could lead to significant improvements in diagnosis, monitoring, initial treatment decisions and follow-up in autoimmune and inflammatory diseases. Mass cytometry provides measurement of over 40 simultaneous cellular parameters at single-cell resolution, and therefore holds immense potential to evaluate complex cellular systems and for high-dimensional single-cell analysis. The high dimensionality of mass cytometry provides better coverage of immune populations dynamics, with sufficient power to identify rare cell types compared to flow cytometry. In this comprehensive review, we explore how mass cytometry findings contributed in the past decade to a deeper understanding of the cellular actors involved in systemic auto-immune and auto-inflammatory diseases with their respective therapeutic and prognostic impact. We also delve into the bioinformatical approaches applied to mass cytometry to analyze the high volumes of data generated, as well as the impact of the use of complementary single cell RNA sequencing, and their spatial modalities. Our analysis highlights the fact that mass cytometry captures major information on cell populations providing insights on the complex pathogenesis of autoimmune diseases. Future research designs could include mass cytometry findings in association to other -omics to stratify patients in adequate therapeutic arms and provide advancements in personalized therapies in the field of auto-immune and inflammatory diseases.

## Graphical Abstract

Interest of using mass cytometry for systemic autoimmune and inflammatory diseases immunophenotyping.

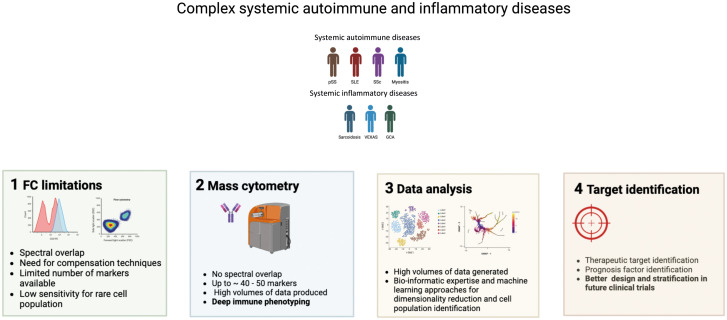

## Highlights

- Mass cytometry has revolutionized immunophenotyping, especially for complex and simultaneous characterization of diverse immune subsets involved in autoimmunity.
- The interpretation of mass cytometry findings is complex due to the high volume of data generated and requires bioinformatical approaches including machine learning.
- Mass cytometry contributes to reveal the heterogeneity present in autoimmune and inflammatory diseases, uncovering targets with therapeutic and prognostic impact.

## Introduction

Auto-immune and inflammatory diseases are heterogenous in their clinical manifestations and prognosis, including between individuals presenting with the same disease. Advances in single-cell technologies are revolutionizing the field of immunology, revealing an underappreciated complexity of immune cell subsets, types, with different phenotypes and function. Understanding the immunological alterations involved in the pathogenesis of autoimmune and autoinflammatory provides valuable insights into the different clinical presentations and treatment responses in these conditions. Single-cell technologies, in particular mass cytometry, have potential to lead to significant improvements in diagnosis, monitoring, initial treatment decisions and/or follow-up in autoimmune and inflammatory diseases. Flow cytometry is known as a key tool to study immune system actors’ implications in various pathological conditions. However, the use of flow cytometry remains highly susceptible to variations with technological limitations regarding sample preparation, and a limited number of usable markers due to spectral overlap, autofluorescence, and instrument constraints (1). As a result, flow cytometry requires large sample sizes (multiple tubes with different antibody panels) for coverage of diverse immune subsets.

Mass cytometry is an -omic approach which embodies the fusion of two technologies: mass spectrometry and flow cytometry. This approach uses elemental mass spectrometry to detect metal-conjugated antibodies that are bound intracellularly or extracellularly to antigens of interest on single cells (2). In mass cytometry (or cytometry by Time of Flight, CyTOF) and Imaging Mass Cytometry (IMC), the fluorescent labels are replaced with heavy metal ions. These heavy metal ions are not normally present in biological specimens and produce very distinctive mass peaks when detected, which allows for more markers to be measured in a single analysis using mass cytometry. By incorporating a large number of parameters into single stains, both CyTOF and IMC significantly increase the ability to evaluate complex immunological data from limited sample sizes to better understand biological systems, response to therapy and disease signatures (3, 4).

CyTOF has the potential to help characterize immune cell signatures and subtypes linked to clinical outcomes, treatment responses, response to vaccines and disease relapse prediction with high resolution. Mass cytometry provides measurement of over 40 simultaneous cellular parameters at single-cell resolution, and therefore has great potential to evaluate complex cellular systems and for high-dimensional single-cell analysis. Mass cytometry has led to the discovery of disease-associated immunological dysregulations in different fields such as cancer and autoimmunity. The identification of functional changes could guide subsequent therapy and ultimately predict therapeutic outcomes and could be applied to an array of diseases. Both CyTOF and IMC have potential for translational clinical application. In the context of breast cancer, the identification of multiplex spatial cellular relationships in a large cohort of breast tumor samples has helped improve histopathological classification. Single cell pathology groups assessed using IMC were superior to standard histopathological grading criteria based on hormone receptor and human epidermal growth factor 2 expression to predict clinical outcomes (5).

During mass cytometry-generated data analysis, machine learning (ML) algorithms are implemented to detect predicting patterns and immune signatures. Specifically, supervised, and unsupervised ML algorithms are widely used for data hierarchical clustering and identification of differentially abundant clusters.

The level of evidence for mass cytometry is recent but has seen an exponential rise in publications for revealing many facets of cellular behavior from a single experiment.

In this review, we explore how mass cytometry has contributed in the past fourteen years to a deeper understanding of the immune cell profiles and heterogeneity present in systemic autoimmune and inflammatory diseases, with their respective therapeutic and prognostic impact. We also discuss how RNA-seq and single-cell RNA seq (scRNA-seq) are being applied to study immune cell subsets in the blood, target organs and tissue-resident cells. Our objectives are: **i/**to summarize how CyTOF, a high-dimensional single-cell tool, allows the identification of complex cellular features in patients with immune mediated systemic diseases, in mirror with other single cell analyses **ii/**to provide insights on the complex pathological mechanisms of autoimmune diseases and their correlation with disease activity and clinical improvement, as well as the discovery of cellular signatures with diagnostic and therapeutic potential across various systemic autoimmune diseases. **iii/**We also provide insights on the ML techniques applied to mass cytometry for the analysis of the high volumes of data generated.

## Computational analysis

Mass cytometry is a multiparametric technology that helps depict an exhaustive immune phenotype. The vast amount of data generated helps gain better insight into immune polarization, cellular differentiation and intracellular signaling in various pathological conditions. The analysis of large volumes of generated data is impossible manually and necessitates the application machine learning algorithms which help discard the subjective aspect of manual gating used in conventional flow cytometry. The main algorithms used in the articles presented in this review include Spanning-tree Progression Analysis of Density-normalized Events (SPADE), self-organizing maps (FlowSOM), cluster identification, and characterization and regression (CITRUS). T distributed stochastic neighbor embedding (t-SNE) and uniform manifold approximation and projection (UMAP) were the two main algorithms used for dimensionality reduction.

The SPADE computational approach is a hierarchical clustering algorithm developed in 2011 (6). The workflow involves a first step of downsampling the data to reduce computational cost, followed by agglomerative clustering of cells based on their phenotypes. This tool helps represent different cell subsets in the form on minimal spanning trees (MSTs), which are a visual representation of clusters’ phenotypical resemblance. MSTs are built in a way that the sum of the weights of the branches is minimal, allowing the nodes to be connected to the ones they are the most similar to (6). One main limitation of the SPADE algorithm involves the slowness of execution, especially when involving larger datasets.

FlowSOM is an algorithm based on the generation of self-organizing maps (SOMs) using an unsupervised approach and a specific type of neural network. The analytical workflow involves 4 different steps, starting with reading the data and building SOMs, followed by building an MST and performing metaclustering. The generation of groups of clusters – metaclusters - help annotate the behavior of specific cell populations and better visualization. Cluster nodes, like in SPADE algorithm, are represented as an MST. The execution time however is faster when compared to SPADE (7).

CITRUS is a machine learning algorithm also widely used to assess cell clusters. It also uses a system based on the hierarchization of cells according to phenotypical resemblance. Statistical relevance is automatically assessed; however, statistical relevance as p-values must be calculated using a different system. Data can be visualized using radial hierarchy trees, in which two parent clusters give “birth” to clusters who represent subsets of the parent cluster (8).

ViSNE is a tool dedicated to CyTOF data analysis, that permits dimension reduction using the t-distributed stochastic neighbor embedding (t-SNE) algorithm. The result is a two-dimensional plot with a display of cells according to their phenotypical resemblance. PhenoGraph also uses the t-SNE algorithm, but contrary to ViSNE, stratifies every event into subpopulations (cell clusters) (9).

Imaging mass cytometry is an advanced tool to assess the immune phenotype in the context of various diseases, with an additional spatial element and the possibility of visualizing direct cell-cell interactions. However, direct comparisons between CyTOF performed on PBMC and IMC performed on tissue remains technically challenging, in part due to the difference in the type of cells analyzed (PBMCs or whole blood with CyTOF, vs formalin-fixed, paraffin embedded tissues in IMC). IMC also presents a somewhat lower dimensionality owing to the lower number of antibodies useable in a single staining protocol. Furthermore, bioinformatical expertise is also needed to assess to quantify and assess the behavior of various cell clusters. Cell segmentation is usually performed using pre-trained deep-learning algorithms based on neural networks, like the Mesmer algorithm developed by Greenwald et al. (10).

## Methods

A systematic search on Medline databases from January 2010 to March 2024 was carried out to summarize the results of published studies on mass cytometry and IMC features of autoimmune diseases.

Different combinations of the following terms were used: “mass cytometry”, “CyTOF”, “imaging mass cytometry”, AND “autoimmunity”, “autoimmune disease”, “connective tissue diseases”, “lupus”, “Sjögren syndrome”, “systemic sclerosis”, “myositis”, “sarcoidosis”, “vasculitis”, “VEXAS syndrome”.

## Systemic autoimmune diseases

**Figure 1:**
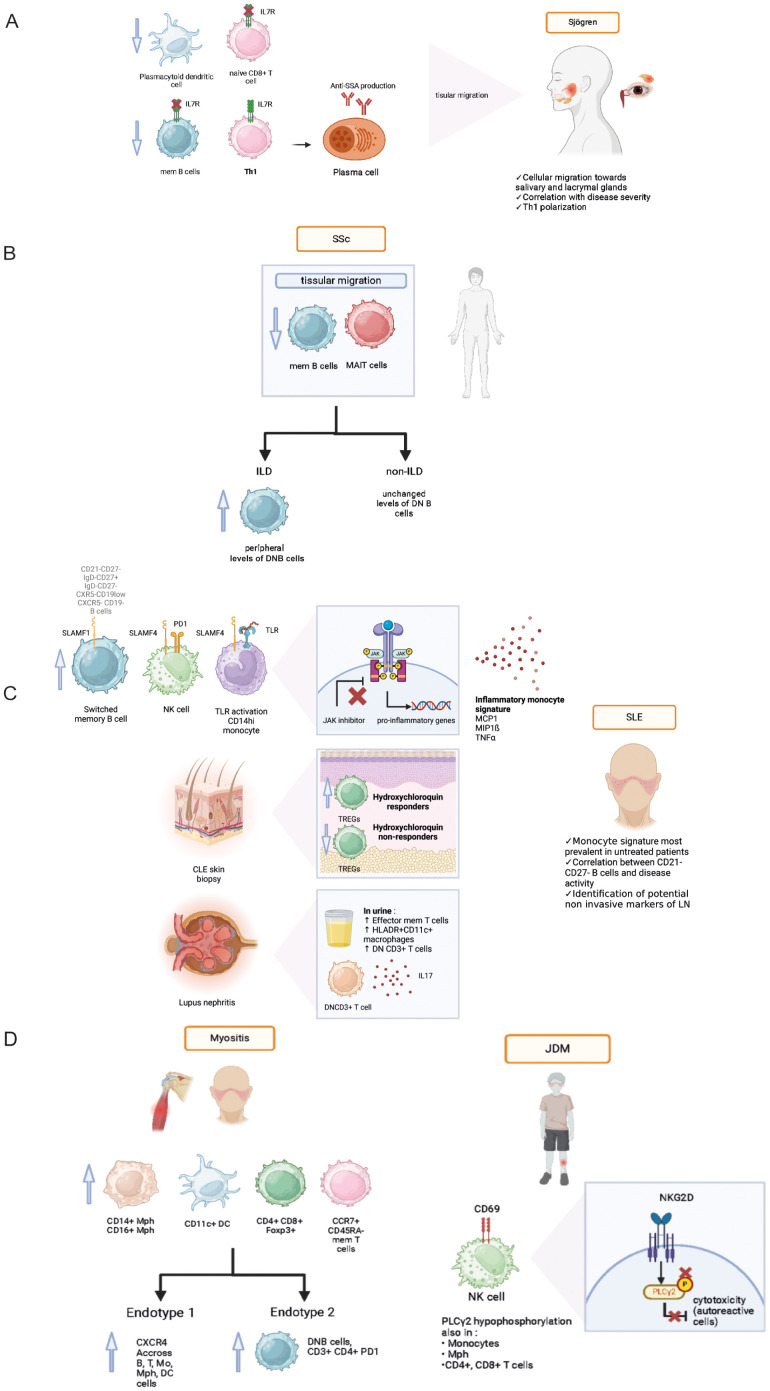
Pathophysiological data acquired from mass cytometry analyses in various systemic autoimmune diseases: Sjögren syndrome **(A)**, Systemic sclerosis **(B)**, Systemic lupus erythematosus **(C)**, Idiopathic inflammatory myopathies and juvenile dermatomyositis **(D)**. Th1, T helper 1, IL7R, IL7 receptor; SLAMF, signaling lymphocytic activation molecule family; SLE, systemic lupus erythematosus; SLEDAI, systemic lupus erythematosus disease activity index, PD1, programmed death cell 1; TLR, toll-like receptor; JDM, juvenile dermatomyositis; NKG2D, Natural killer group 2D; mem T cell, memory T cell; mem B cell, memory B cell; Mph, macrophage; DC, dendritic cell; DN B, double negative B cell; MAIT, mucosal-associated invariant T cell; CXCR4, C-X-C chemokine receptor type 4; ILD, interstitial lung disease.

Systemic autoimmune diseases are characterized by their complexity and a high number of immunological actors involved in their physiopathology. Mass cytometry has helped identify clusters of cells associated with diseases and prognosis (**Table 1** and **Figure 1**).

**Table 1:** Mass cytometry in Sjögren syndrome and systemic sclerosis.

Disease	1^st^ author, year	Aim	Number of patients	Technical approach	Abnormalities in cell distribution	Complementary transcriptomic analysis	Impact (prognosis/therapeutic)	Take home message	Algorithm
**pSS**	Minguenau, 2016 (15)	Identify new cellular biomarkers and therapeutic targets in patients with pSS.	49 pSS 45 HC	CyTOF on PBMC	**In pSS vs HC**: ↓* circulating CD4 ↓ mB cells ↓ pDC ↑^†^ activated CD4, CD8, plasmablasts and plasma cells	*Standalone CyTOF analysis*	Cellular signature stratified patents into groups with distinct clinical and pathological features. High ESSDAI: more severe CD4 lymphopenia and increase in activated CD4 and CD8.	Identification of a **6-cell disease signature** in the blood.	ViSNE
	Desvaux, 2023 (18)	Perform a deep characterization of the IL7/IL7R pathway in pSS	39 pSS (PBMC) 21 pSS (MSG) 30 HC 2 MALT 5 sicca syndromes	CyTOF on PBMC IMC on MSG	**In pSS vs HC:** ↓IL7R expression on CD4, CD8+ naïve T cells, TCM, TEM ↑**Th1 expressing IL7R**	Bulk RNA-seq in whole blood and in salivary glands. • 49 gene IL7 signature in pSS • Following IL7 stimulation: ↑KZF4, KIAA0040, PGAP1 and SOS1, associated with anti-SSA/Ro and ESSDAI.	Possibility to target IL7 pathway in pSS.	High expression of IL7R on Th1 cells indicates a strong Th1 activity in pSS. Gene expression upregulation following IL7 stimulation was associated with SSA/Ro positiveness and disease activity. IL7 was associated with immune infiltration severity in MSG.	SPADE
**pSS, SLE, SSc**	Van Der Kroef, 2020 (19)	Assess immune differences between SSc, pSS and SLE	Identification cohort: SSc: 19, SLE: 13, pSS: 8 44 HC Replication cohort: SSc: 69 SLE: 18 pSS: 15	CyTOF on PBMC	**In SSc**: ↓ CD56hi NK cells ↓ IgD+ mB cells; ↑ cMo **In SLE**: ↑ plasmablasts ↓ IgM B cells; ↓ IgD+ MBCs. **In pSS**: ↓ IgM+ B cells. **In pSS + SSc + SLE:** ↓ pDC	*Standalone CyTOF analysis*	**Prognosis:** Correlation between monocyte frequency and skin fibrosis, ILD in SSc. Correlation between ↓ in IgM B cells and SLEDAI in SLE.	Some unique changes in cell subsets in some diseases but too low or discriminative power for diagnostic purposes between AIDs. Common feature: decrease in pDC in AIDs, with common IFN signature. pDC are major producers of IFNα, with possible migration to local tissue for IFN I production	t-SNE, Euclidean distance and minimal variance methods, Quantile normalization
**pSS**	**Sarkar** (16)	Aberrant signaling of immune cells in Sjögren’s syndrome patient subgroups upon interferon stimulation	pSS SSA+: n=8 pSS SSA-: n=8 HC: n=8	CyTOF on PBMC	**In pSS:** ↑HLA-DR expression in pSS group, predominantly in mBC subset **In pSS-SSA+:** ↑ B cells, T cells, NKT cells in pSS SSA+ subgroup ↑ CD38 on mBC, cDCs and conventional monocytes ↑STAT1 Y701 following IFNg stimulation in pSS SSA+ cDC	*Standalone CyTOF analysis*	Possible implication of STAT1 Y701 pathway in the interferon signalling in pSS patients	A shift in tyrosine kinase activation profile towards STAT7701, away from STAT3, STAT4, STAT5 is observed in pSS patients. Increased STAT1 Y701 following IFN stimulation was observed in pSS patients, as well as differential leucocyte profile among pSS SSA+ subset.	
**Systemic sclerosis**	Paleja, 2020 (20)	Immunophenotyping of SSc patients.	SSc: 20 HC: 10	CyTOF: PBMC Complementary RNA-seq	**In SSc** : ↓ MAIT cells (TCR Va 7.2 CD161+) ↓ mB cells	*Standalone CyTOF analysis*	MAIT and CD4 cells significantly decreased in ILD compared to patients without ILD	Identification of cell subtypes changes depending on the presence of visceral involvement associated with high risk (ILD).	tSNE Correlation networks
	Wilfong, 2022 (21)	To investigate the potential role of a B cell population as a cellular biomarker for SSc-ILD.	34 patients with SSc-ILD 14 SSc without ILD 25 HC	CyTOF: PBMC	**In SSc-ILD vs SSC without ILD, and SSc-ILD vs HC**: ↑ CD19+ CD21 lo/neg cells	*Standalone CyTOF analysis*	Possible therapeutic target: CD19+ CD21low/neg cells.	CD21 lo/neg B cells are elevated in SSc ILD patients, but NOT in SSc patients without ILD, nor in healthy controls. CD21 lo/neg B cells are possibly potent CD4+ T cells activators, previously described as Th17 activators in mice.	tSNE

cMo, conventional monocytes; CyTOF, cytometry by time of flight; ESSDAI, EULAR Sjögren’s syndrome (SS) disease activity index; HC, healthy controls; ILD, interstitial lung disease; IMC, imaging mass cytometry; IPF, idiopathic pulmonary fibrosis; MAIT, mucosal-associated invariant T cells; MALT, mucosal-associated lymphoid tissue; mB, memory B cells; MSG, minor salivary gland; NK, natural killer; pDC, primary dendritic cells; pSS, primary Sjögren syndrome; PBMC, peripheral blood mononuclear cells; SSc, systemic sclerosis; TCM, T central memory; TEM, T effector memory; t-distributed stochastic neighbor embedding; ViSNE, Visualization of t-Distributed Stochastic Neighbor Embedding. *↓ signifies a decrease in a specific cell population. ^†^↑ signifies an increase in a specific cell population.

### Primary Sjögren syndrome (pSS)

Primary Sjögren’s syndrome (pSS) is a systemic autoimmune disease, characterized by mononuclear cell infiltrates in the salivary and lacrimal glands, leading to glandular atrophy and dryness. Patient heterogeneity and lack of knowledge regarding its pathogenesis makes pSS a difficult disease to manage, and explains in part the absence of validated treatment. Moreover, symptom burden and immunomodulatory medication do not correlate with pSS end-organ or laboratory abnormalities (11, 12). Robust ML methods exist to stratify pSS patients based on five common symptoms associated with the disease (13). Symptom-based stratification of pSS patients revealed distinct pathobiological endotypes with different responses to immunomodulatory treatments (14). Heterogeneity is a major obstacle to develop effective treatments for pSS patients, motivating authors to perform an extensive landscape of the immune cells involved in pSS physiopathology using mass cytometry. Mingueneau et al. identified a 6-cell disease signature defined by decreased numbers of memory B lymphocytes, plasmacytoid dendritic cells (pDCs) numbers, increased representation of activated CD4+, CD8+ T cells and plasmablasts (**Table 1**). The cellular signature helped separate the cohort into groups with distinct clinical and pathological features. Patients with higher EULAR Sjögren syndrome disease activity indexes (ESSDAI) were associated with a higher proportion of activated CD4+ and CD8+, and more pronounced CD4+ lymphopenia (15).

Sarkar et al. showed that IFNα2b stimulation results in an increased signaling through phosphorylated signal transducer and activator of transcription protein 1 (pSTAT1), a major actor involved in cytokine production. This pSTAT1 production was observed in most cell types of pSS patients compared to controls. IFNγ stimulation resulted in significantly increased pSTAT1 induction in conventional DCs, classical and non-classical monocytes in the pSS patients (16), highlighting a high sensitivity to inflammatory stimuli. Most of the observed differences were more pronounced in the SSA+ subgroup, suggesting that these patients may benefit from therapies targeting these pathways (**Table 1**).

IL-7 cytokine is involved in B and T cell lymphopoiesis and T cell maturation. The increased expression of its receptor in epithelial tissue could participate to chronic inflammation and subsequent symptoms. Studies had previously shown increased IL-7 levels, associated high EULAR Sjogren syndrome disease activity index (17). Desvaux et al. further investigated the role IL-7/IL-7R pathway in pSS patients (18) using a multimodal approach. Initially performing bulk RNA-seq, they highlighted the IL7 axis activation at the transcriptomic level in the whole blood of pSS patients as well as salivary glands. Performing mass cytometry helped confirm IL7/IL7R axis implication in relevant circulating and target-organ cell populations. IL-7R was mainly found on epithelial cells, CD4+ and CD8+ T cells, switched memory B cells, double negative (DNB, CD21- CD27-) B cells and M1 macrophages (**Figure 1A**). The authors found a higher expression of IL7-R at the surface of T helper 1 (Th1) cells, confirming the role of Th1-polarized immunity in pSS. Anti-IL-7R targeted therapy could be an interesting therapeutic approach in pSS patients with a high IL-7 axis involvement (**Table 1**).

### Systemic sclerosis (SSc)

Systemic sclerosis is a heterogeneous autoimmune disease associating fibrosis, inflammation, and microvascular disease. Before the onset of skin fibrosis and other organ involvement, patient with Raynaud’s phenomenon and positivity for SSc-specific autoantibodies and/or typical nailfold capillaroscopy abnormalities are considered early SSc patients. Van der Kroef et al. demonstrated that increased frequency of intermediate and non-classical monocytes was specific for early and definite SSc (19).

Paleja et al. used a CyTOF panel composed of cell surface and intracellular markers to comprehensively characterize peripheral immune cell composition in SSc patients and healthy controls (HC) (20). Unsupervised clustering revealed disease-specific alterations in PBMCs from SSc patients with a decrease in memory B cell and a concomitant increase in naive B cells, and significant reduction of mucosal associated innate T lymphocytes (MAIT cells) and DN (CD4^−^CD8^−^) T cells in SSc patients (**Figure 1B**). Correlation analysis of mass cytometry data revealed an inverse correlation between programmed death cell 1 (PD-1) expression and the frequency of MAIT cells in SSc patients compared to healthy controls. In this study, the combination of high-dimensionality data identified cell subsets reminiscent of functionally adapted, exhausted T cells in response to chronic stimulation (**Table 1**).

Interstitial lung disease (ILD) is a major cause of mortality and morbidity in SSc, primarily due to associated pulmonary hypertension and subsequent right ventricle dysfunction. Wilfong et al. (21) identified the presence of DNB CD21^-/low^ cells who were elevated in SSc patients presenting with ILD compared to SSc controls, and could thus serve as a potential biomarker.

### Systemic lupus erythematosus (SLE)

SLE is an auto-immune disease that affects multiple organs with an unpredictable course, and can lead to irreversible visceral damage when untreated. Current diagnostic strategic methods are insufficient to predict the severity and course of the disease, the occurrence of a flare and treatment response. Identifying early-stage SLE presents a challenge as initial symptoms are often non-specific and autoantibody production alone is not sufficient to establish diagnosis. Sasaki et al. (22) identified several cell populations expanded during early-stage SLE: antibody secreting plasmablasts, helper T cells (Tph), and T follicular helper (Tfh) cells were identified as potential biomarkers for early SLE. These populations could potentially serve as biomarkers and early therapeutic targets (**Figure 1C**).

Evidence points toward a polarization of the immune phenotype depending on disease activity. A deeper understanding of SLE physiopathology can help identify prognosis factors. Increased levels of DN B cells were described in the context of SLE (23). In a pediatric cohort of childhood onset SLE (cSLE), Baxter et al. (24) revealed the expansion of an extrafollicular B cell and T peripheral helper (Tph) population, as well as a correlation between the presence of cytokine producing B cells and disease activity. In this study of new-onset cSLE, the authors conducted a complementary bulk RNA-seq analysis, which validated previously published findings of an increased peripheral plasmablast signature and a type I IFN signature. The strength of this integrated approach relied on its use of the same patient cohort, allowing for a direct comparison to abnormalities observed using mass cytometry. Additionally, a novel population of plasmablast-like CXCR5- CD19^low^ cells was identified in a cohort of adult patients affected with chronic SLE using mass cytometry (25). Following these findings, using a targeted RNA-seq approach performed on FC-sorted B cells, the authors identified the expression of transcription factors involved in plasma cell and plasmablasts survival (*IRF4, PRDM1, XBP1, EZH2)*. Other cell populations were associated with SLE. Kajihara et al. (26) identified 5 clusters based on the proliferative activity of 5 immune cell populations: conventional monocytes (cMo), CD8+ effector memory T cells, CXCR5- non-B cells, CXCR5- DN B cells and plasma cells, with varying associated disease activity index score (SLEDAI).

SLE also presents therapeutic challenges. To this day, only one targeted biotherapy has been validated: Belimumab, an anti-BAFF monoclonal antibody targeting the cytokine essential for B cell homeostasis. The results of Ramskold et al. showed B cell subset alterations in responders versus non-responders following Belimumab administration in SLE patients (27). The authors suggested that evaluation of B cell counts might be useful prior to initiation of Belimumab treatment, as high baseline B cell counts were associated with unfavorable outcome. In another study, Belimumab was also found to have an impact on T cell populations with an increase in circulating anti-inflammatory regulatory T cells (TREGs) compared to untreated SLE patients (**Figure 1C**) (28).

Interferon and Janus kinase (JAK) – STAT pathways have been previously associated with SLE physiopathology in conventional cytometry and transcriptomic studies. Yiu et al. showed that high interferon signature leads to increased STAT1/3/5 phosphorylation in peripheral blood circulating mononuclear cells (PBMCs) from SLE patients studied using single cell mass cytometry. Patients were divided into interferon (IFN) high and IFN-low groups based on their transcriptomic profiles. IFN-high patients displayed higher proportions of circulating B and NK cells, and cytokine stimulation was associated with higher activation of STAT1/3/5 intracellular pathways. In another study, O’Gorman et al. (29) identified the presence of a specific monocytic signature (MCP-1+, MIP-1ß, TNFα) in a population of untreated SLE patients stimulated with TLR (toll-like receptor) agonists. The signature was also identified in a population of pediatric SLE patients, and was suppressed by JAK inhibitor treatment (30). These findings provide further support for the development of JAK inhibitor therapies in the context of SLE (31).

New potential targeted therapies were identified following deep immune phenotyping using mass cytometry. Humbel et al. (32) unveiled an increased expression of molecules belonging to the signaling lymphocytic activation family (SLAMF) on memory B cells, monocytes, NK cells and Tfh in SLE compared to other auto-immune diseases (**Figure 1C**). Targeting SLAMF in SLE with elotozumab, an anti-SLAMF7 antibody, restored NK cytotoxic cell functions in SLE patients, priming them to kill antibody-secreting plasma cells *in vitro*, making NK cells a target of interest in SLE management (33). Rincon-Arevalo et al. showed an increased frequency of CD11c+ B cells carrying CD21- CD27- phenotype in SLE patients (34). In addition, the authors found other markers of B cells activation such as reduced CD21, and increased expression of activation (CD69, CD86), proliferation (Ki67), survival (CD137) and co-inhibitory (PD1, PDL1) markers (**Table 2**).

**Table 2:** Mass cytometry in lupus: PBMC and whole blood analysis.

Disease	1^st^ author, year	Aim	Number of patients	Technical approach	Abnormalities in cell distribution	Complementary transcriptomic analysis	Impact (prognostic/therapeutic)	Take home message	Algorithm
**Lupus**	Sasaki, 2022 (22)	Investigate the immune cell profiles in SLE, and identify longitudinal changes over time	9 early SLE 15 established SLE 14 HC	CyTOF: PBMC	**In early SLE** ↑* Tph, Tfh ↑ ICOS+ Ki67+ CD8+ T cells ↑ Ki67 TREGS ↑ CD19int, Ki67hi plasmablasts ↑ PU.1high Ki67 hi monocyte ↑ Plasmablasts, ABCs **In established SLE** ↑ Plasmablasts, ABCs, Tph	*Standalone CyTOF analysis*	**Prognosis impact:** Impact of the elevation of cell populations on the early diagnosis of preclinical lupus	• Several cell populations expanded in the early stages of SLE with ↑ of Ki67. • Antibody secreting plasmablasts, Th cells, Tfh, Tph could be biomarkers for early SLE • CXCL13 were positively corelated with several of the expanded cell populations in early SLE	tSNE, flowSOM
	Horisberger, 2022 (23)	Establish cellular biomarkers in SLE, by studying B cell populations	30 SLE in CyTOF 63 SLE in FC (= validation cohort), compared to 14 pSS, 14 sarcoidosis, and 39 HC	CyTOF: PBMC FC for validation	**In SLE** ↑ CD21- CD27- B cells	*Standalone CyTOF analysis*	**Prognosis**: Association between CD21- CD27- populations and disease activity, independent from treatment and serological biomarkers. CD21- CD27- B cells are elevated in SLE patients and correlated to disease activity.	CD21- CD27- B cells are elevated in SLE patients and correlated to disease activity. This elevation is specific to SLE compared to pSS, sarcoidosis and HC (comparison performed in FC).	tSNE, FlowSOM
	Baxter, 2023 (24)	Immunophenotyping, and understand the modest efficacy of targeted therapies in childhood onset SLE	24 treatment naïve childhood-onset SLE (cSLE) 30 age sex matched HC	CyTOF: Whole blood	**In SLE**: ↑ Extrafollicular B cell expansion ↑ DN2 B cells CD11c+ CD27- IgD- ↑ Bnd2 cells, Plasmablasts ↑ Tph, Tfh, CD8+	Bulk RNA-seq performed on whole blood. • Confirmation of increased peripheral plasmablast signature • Confirmation of increased IFN I signature	**Prognosis**: Correlation between presence of cytokine producing B cells and disease activity	• Evidence of extrafollicular B cell and peripheral T helper cell expansion confirmed in paediatric populations. • Frequency of the B cells correlated with cTph and cTfh in children. • Possibility of targeting novel B cell differentiation and/or activation pathways identified using transcriptomic analysis.	Lance William dissimilarity update
	Szelinski, 2022 (25)	Characterize the role of double negative IgD-CD27- B cells in SLE	24 SLE 18 HC	CyTOF: PBMC FC validation: 28 SLE and 16 HC + Targeted RNA-Seq analysis	**In SLE**: ↑IgD-CD27+ B cells ↑IgD-CD27- atypical mB cells **Among DN and switched mB cells:** ↓^†^ BTLA ↑ CXCR5+CD19 intermediate ↑ CXCR5-CD19low (novel) ↑CXCR5-CD19high	Targeted RNA-seq performed on FC-sorted B cell populations. • ↑ IRF4, PRDM1, XBP1, EZH2 in both plasmablasts and DNB^low^ cells	**Therapeutic impact**: Co-targeting the CXCR5-CD19low B cell subset (= plasmablasts precursor) may have therapeutic value in SLE	• SLE associated with 2 novel CXCR5-CD19low subsets (mB^low^ and DNB^low^), with the following characteristics: • Plasmablasts-like transcriptional in DNB^low^ cells programming (IRF4, PRDM1, XBP1, EZH2 expression revealed using RNA-seq) • Diminished B cell receptor responsiveness • Phenotype: CD38+ CD95, CD71	UMAP
	Kajihara, 2022 (26)	To search for a link between immune cell populations and clinical phenotypes of SLE, and compare expression of Ki67 among them	28 SLE 15 HC	CyTOF: PBMC	**SLEDAI** was positively correlated with cMo and plasma cells **C4** and **anti**-**DNA** were positively correlated with CXCR5- DNB cells (CD27-, IgD-) and CXCR5 nB	*Standalone CyTOF analysis*	**Prognosis**: Patients changed clusters following immunosuppressive therapy initiation	5 clusters based on proliferative activity of 5 immune cell populations: **conv monocytes, CD8Tem, CXCR5- nB, CXCR5- DNB, plasma cells** • **Cluster 1:** low proliferation in 5/5 cell populations • **Cluster 2: high SLEDAI and anti-DNA**, with high proliferation in cMo and PC + • **Cluster 3: Kidney involvement and moderate SLEDAI;** high proliferation in in CXCR5- nB and CXCR5- DNB cells + • **Cluster 4: low SLEDAI,** high CD8Tem and cutaneous rash • **Cluster 5: mild SLEDAI**, high proliferation in cMo and PC, fever	k-means clustering
	Ramsköld, 2019 (27)	Study the alterations in leukocyte population following Belimumab administration	23 patients with moderately active SLE, treated with Belimumab 19 RR multiple sclerosis 10 HC	CyTOF: PBMC	**In SLE patients:** ↑ CD57+ B cells (also in RRMS) **Following Belimumab:** ↓ naive B cells ↓ DN mB cells ↓ ABCs, CD11c+, CD21-, CD27 low	*Standalone CyTOF analysis*	**Prognosis**: High baseline B cell counts predict unfavourable treatment outcomes. Evaluation of B cell counts might be useful prior to treatment initiation. **Therapeutic**: Effect of Belimumab on DN B cells, naive B cells and mB cells	• Belimumab provokes rapid deterioration effect on B cells at early stages, but later stage B cells showed delayed to no response. • At baseline in SLE patients: ↑ of a novel B cell population: CD57+ B cells.	ACCENSE, PhenoGraph
	Maeda, 2023 (28)	To study the impact of belimumab on T cell immune profiling in SLE	22 SLE patients treated with Belimumab 20 SLE controls who did not receive Belimumab	CyTOF: PBMC	**Among Belimumab treated patients** ↑ TREG cluster ↑ functional TREG ↓ Th17/fTREG ratio ↓ pTh cell/fTREG ratio ↑ central memory activated T cells ICOS + CD28+ CD38+	*Standalone CyTOF analysis*	**Prognosis: Belimumab treatment:** ↑ CH50 and C4. T cell population resistant to Belimumab: ICOS+ CD28+ CD38+ central memory T cells. **Therapeutic:** Belimumab linked to a ↓ in daily prednisolone use.	• Belimumab has an effect on T cell immune tolerance: increase and functional variations in TREG population. • Belimumab permits a favourable balance of Th17/TREG ratio • Activated central mB cells (CD38+, ICOS+, CD28+) are a potential novel target to obtain immune remission under Belimumab	FlowSOM
	O'Gorman, 2015 (29)	Characterize TLR activation across immune cell subsets in subjects with SLE	SLE: 17 (newly diagnosed, untreated) HC: 17	CyTOF: PBMC Complementary FC with JAKi experiments	**In SLE vs HC:** In CD14^hi^ monocytes: ↑MCP1, MIP-1ß, TNFα	*Standalone CyTOF analysis*	**Prognostic impact**: MCP-1 previously associated with lupus nephritis in humans (56)	Untreated SLE patients present an activated monocytic signature (MCP-1 = CCL2, MIP-1ß, TNFα), the most prominent being MCP-1.	CITRUS
	O'Gorman, 2017 (30)	Analyse the immune landscape of SLE in paediatric patients	10 newly diagnosed untreated paediatric SLE 10 age and gender matched HC	CyTOF: PBMC	**In CD14hi Monocytes of SLE patients:** ↑MCP1, Mip1ß	*Standalone CyTOF analysis*	**Prognosis**: Fluctuation of monocyte cytokine signature with clinical disease activity (SLEDAI) **Therapeutic impact**: JAK1/JAK2 inhibitors provoked inhibition of monocyte cytokine signature	• Identification of a distinct CD14hi monocyte cytokine signature • The monocyte signature was present in every patient, even with different clinical presentations • Reproduction of monocyte signature following SLE plasma administration to HC blood. • Inhibition of monocyte signature using JAK1/JAK2inhibitors, but only partially with IFN type I receptor blockade	CITRUS, ViSNE
	Yiu, 2022 (31)	To investigate the IFN signature in SLE using CyTOF	25 SLE (divided into IFN High and IFN Low) 9 HC For intracellular cytokine production assessment: 31 SLE, 17 HCs	CyTOF: PBMC	**In IFN High patients, compared to IFN Low:** ↑ % of B cells ↓ % of NK cells **In SLE vs HC:** ↓ % TREGS (not observed when comparing SLE IFN High vs SLE IFN Low)	*Standalone CyTOF analysis*	**Therapeutic impact**: development of JAK inhibitors in SLE.	Differences in cellular phenotypes of SLE patients with IFN High and IFN Low profiles: • IFN High patients had higher phosphorylation of STAT1/3/5 following cytokine stimulation • IFN High patients also had increased phosphorylation of non-canonical STAT proteins	FlowSOM
	Humbel, 2022 (32)	To identify a SLE specific immune signature based on SLAMF expression	38 SLE HC and Auto immune diseases controls (pSS, 10, sarcoidosis 10, SSc 10)	CyTOF: PBMC FC validation	**In SLE compared to other AIDs:** ↑ SLAMF1 on switched mB cells ↑ SLAMF4 on monocytes and on NK cells ↑ SLAMF3, 5, 6 on mB cells ↑ SLAMF+ Tfh	*Standalone CyTOF analysis*	• Inverse correlation between SLAMF4+ NK cells and monocytes and SLEDAI • SLAMF1 CD4+ TEM cells were correlated with disease activity	Presence of a specific SLAMF signature in SLE patients. Increased SLAMF1+ B cells and SLAMF in SLE compared to other auto-immune diseases	FlowSOM
	Humbel, 2021 (33)	To study the phenotype of NK cells in treated SLE patients	44S LE HC	CyTOF: PBMC	**In NK cells in SLE** ↑ CD38 ↑ PD1Lack of SLAMF7 and SLAMF1 following stimulation.	*Standalone CyTOF analysis*	**Therapeutic impact:** Targeting CD38, SLAMF1, SLAMF7 in SLE	NK cells expressed higher levels of CD38 compared to HC • NK cells are dysfunctional in SLE: lack of SLAMF1 and SLAMF7 following activation • Treatment with elotuzumab (SLAMF7 ligand) or daratumumab restored NK cell function.	tSNE
	Rincon-Arevalo, 2021 (34)	To study CD11c+ B cells in SLE, pSS and HD	27 SLE 22 pSS 18 HD	CyTOF: PBMC	**In SLE and pSS compared to HD** ↑CD11c+ B cells, with: • CD21- CD38- ↑Activation (CD69+, CD86+), proliferation (Ki67), prosurvival (CD137) ↑ PD1, PDL1, CTLA4, ICOS, CD86	*Standalone CyTOF analysis*	Insights on SLE physiopathology.	SLE CD11c+ B cells notably presented as CD27- CD38-, and expressed checkpoint molecules. The presence of CD21- B cells expressing activation and checkpoint markers could be linked to an increased extrafollicular B cell activation route in SLE.	tSNE

ABCs, age associated B cells; AID, autoimmune disease; AS, ankylosing spondylitis; Bnd cells, anergic autoreactive B cells; CCL2, chemokine ligand 2; CITRUS, cluster identification, and characterization and regression; CLE, cutaneous systemic lupus erythematosus; cMo, conventional monocytes; cSLE, childhood-onset SLE; CyTOF, cytometry by time of flight (mass cytometry); DNB, double negative (CD21- CD27-) B cells; ESSDAI, EULAR Sjögren’s syndrome (SS) disease activity index; FC, flow cytometry; fTREG, functional TREG; HC, healthy controls; HCQ, hydroxychloroquine IMC, imaging mass cytometry; JAKi, JAK inhibitor; LN, lupus nephritis; MALT, mucosa associated lymphoid tissue; mB cells, memory B cells; MCP-1, monocyte chemoattractant protein 1; MSG, minor salivary glands; nB cells, naïve B cells; NK, natural killer; PBMC, peripheral blood mononuclear cells; SLAMF, signaling lymphocytic activation molecule family; SLE, systemic lupus erythematosus; SLEDAI, systemic lupus erythematosus disease activity index; SPADE, Spanning-tree Progression Analysis of Density-normalized Event; TEM, T effector memory cells; Th, T helper cell; Tfh, T follicular helper; Tph, T peripheral helper; TLR, toll like receptor; TNFR, TNF alpha receptor; TREGs, regulatory T cells; rTNF, recombinant TNF; ViSNE, visualization tool based on t-Distributed Stochastic Neighbour Embedding (t-SNE) algorithm. *: ↑ signifies an increase in a specific cell population; †: ↓ signifies a decrease in a specific cell population.

Cutaneous SLE (CLE) is a frequent manifestation with various degrees of associated comorbidity, and a multifactorial physiopathology which involves environmental factors. Anti-malarials represent the first line treatment but response is unpredictable. Patel et al. (35) compared skin biopsies performed on CLE patients using IMC, stratifying patients according to anti-malarial treatment response. Non-responders displayed a pro-inflammatory profile with diminished levels of TREGs and increased expression of pSTAT3 in CD7+ cells, whereas responders had increased levels of anti-inflammatory γ∂T cells (**Table 3**). These results suggest that non-responders with low TREGs count may respond to low dose IL2 stimulation, whereas non-responders with increased pSTAT3 could probably respond better to JAK inhibitors.

**Table 3:** Lupus tissue analyses.

Disease	1^st^ author, year	Aim	Number of patients	Technical approach	Abnormalities in cell distribution	Impact (prognosis/therapeutic)	Take home message	Algorithm
**Lupus**	Patel, 2022 (35)	Characterize the immune profile of patients with CLE stratified by response to antimalarial treatment	48 skin biopsies of treatment naïve CLE	IMC (skin biopsy)	In HCQ non-responder CLE group: ↓* TREGs ↑^†^ pSTAT3 in CD7+ cells In HCQ responders vs. QC responders ↑ CD4 cell compartment ↑ CD68+ macrophages ↑ gamma delta T cells	**Prognosis**: Different cellular phenotype = different response to antimalarials, immunosuppressant use and global prognosis **Therapeutic**: Non-responders with low TREG cell counts may respond to IL2 low dose. Better response to MMF or JAKi in patients with ↑pSTAT3	Antimalarial is a first line therapy for CLE, but response is unpredictable. Different cellular phenotype observed when stratifying according to HCQ-only response, HCQ and QC response, and non-responders. T cell pathogenesis could play an important role in antimalarial response. HCQ responders had a greater expression of immunosuppressive γ∂T cells.	ClustVis
	Titus, 2023 (36)	Assess the immune landscape of proliferative lupus nephritis (LN) using imaging mass cytometry, and reduce misclassification risk	10 LN with class IV 8 LN with class II 3 HC (minimal change disease and acute tubular necrosis).	CyTOF Exploratory IMC of renal biopsy (n = 1) Validation of IMC results using IHC: 15 LN class IV + 15 LN class V + 10 controls with solitary renal cell carcinoma	In glomeruli and tubulointerstitial regions of proliferative LN: ↑ CD45RO+ HLADR+ memory CD4 and CD8 T cells ↑ CD163+ macrophages	Feasibility study of the application of IMC in LN biopsies	In proliferative LN: • Higher infiltration of memory CD4 and CD8 cells in glomeruli and tubulo-interstitial segments • Higher infiltration of macrophages Macrophages and T cells also predominate in cellular crescents	Mesmer
	Bertolo, 2020 (37)	Identify non-invasive markers (replacing renal biopsy) to predict the response of LN to treatment	13 SLE with biopsy proven LN 6 controls with acute inflammatory renal diseases	CyTOF on urine leukocytes and whole blood	**In urine:** ↑ Activated (CD38+ CD69+) effector memory T cells. Majority was CCR5+, Th1. ↑ HLADR+ CD11c+ macrophages **In both blood and urine:** ↑DN CD3+ T cells with increased IL17 production.	The amount of effector memory T cells was predictive for response to immunotherapy.	• Immune infiltration in urine differs from blood in lupus nephritis. Urine immune cells are probably the reflection of kidney infiltration. • Immune cells in urine display an activated phenotype • Effector memory T cells are potential biomarkers to stratify LN in terms of response to induction therapy • Cluster analysis showed a clear differentiation between proliferative LN, non-proliferative LN and acute inflammatory diseases.	Hierarchical clustering analysis based on Spearman distance and Ward linkage criterion.

CLE, cutaneous lupus erythematosus; ClustVis, clustering visualization tool of multivariate data; DN, double negative; HCQ, hydroxychloroquine; HLA, human leukocyte antigen; IMC, imaging mass cytometry; LN, lupus nephritis; MMF, mycophenolate mofetil; JAKi, janus kinase inhibitor; QC, quinacrine; TREG, regulatory T cell.

Lupus nephritis (LN) can affect up to 50% of SLE patients and is associated with high comorbidity, and risk of chronic kidney disease. Kidney biopsy is an invasive procedure and remains the gold standard for LN diagnosis. Titus et al. studied renal biopsies of proliferative LN compared to acute tubular necrosis controls using IMC. The authors identified an infiltration by CD45RO+ HLADR+ memory CD4+ and CD8+ T cells, associated with CD163+ macrophages (36). Bertolo et al. (37) studied urinary leukocytes using mass cytometry to identify non-invasive biomarkers, and revealed a link between the amount of activated effector memory T cells (Tem) and LN response to immunotherapy (**Figure 1C**).

Despite new comprehensive autoantibody data with novel longitudinal clustering technique in SLE patients predictive of outcome, no integrative approach including immune ML analysis with mass cytometry has been performed to better predict long-term disease activity, organ involvement, treatment requirements and mortality risk.

### Idiopathic inflammatory myositis (IIM)

IIM, also known as myositis, are a group of diverse auto-immune connective tissue diseases presenting with striated muscle inflammation and varying associated clinical manifestations, treatment response and prognosis. In an exploratory analysis, Patel et al. (38) compared dermatomyositis (DM) skin biopsies to healthy controls. The analysis revealed an increased pro-inflammatory infiltrate made of CD14+ CD16+ macrophages, CD11c+ myeloid dendritic cells (mDCs), CD4+ and CD8+ T cells, and CCR7+ CD45RA- memory T cells (**Figure 1D**).

Different subsets of IIM can exhibit very similar clinical presentations. Anti-synthetase syndrome (ASyS) and dermatomyositis (DM) are two entities of IIM which can present with very similar skin lesions, potentially complicating the diagnosis process. In a following analysis, Patel et al. (39)· compared DM-like skin lesions in ASyS patients to skin biopsy samples obtained from DM patients using IMC (**Table 4**). Both DM and ASyS immune infiltrates presented with many overlapping features, but also some disease specific differences. Notably, the authors found a higher infiltration of myeloid cells associated with DM and a higher proportion of macrophages presenting with phosphorylated stimulators of interferon genes (pSTING+) in ASyS patients, with an elevated production of IFNß, TNF, and IL17. An innate immune signature was also unveiled when comparing JDM (juvenile dermatomyositis) skin biopsies to cutaneous lupus, (CLE), with an elevated expression of CD68+ and CD14+ macrophages. IMC analysis also revealed increased cell-cell interactions involving endothelial cells, highlighting the vascular pathology involved in the physiopathology of DM (40). Another study performed on JDM revealed a defect in PLCγ2 phosphorylation, resulting in NK cells dysfunction resulting in decreased cell cytotoxicity and correlating to disease activity (41).

**Table 4:** Mass cytometry in inflammatory idiopathic myositis.

Disease	1^st^ author, year	Aim	Number of patients	Technical approach	Abnormalities in cell distribution	Complementary transcriptomic analysis	Impact (prognosis/therapeutic)	Take home message	Algorithm
**Myositis**	Patel, 2021 (38)	To assess immune phenotype of DM and screen for potential therapeutic targets	10 DM 5 HC	CyTOF: PBMC	**In DM vs. HC**: ↑* CD11c+ mDCs ↑ CD14+ CD16+ Mph ↑ CD4, CD8, Foxp3+ ↑ CCR7+ CD45RA- memory T cells	*Standalone CyTOF analysis*	**Prognosis**: CDASI was correlated with activation of memory T cell infiltrating skin lesions in DM (CD69+ expression) **Therapeutic:** Monocyte - macrophage recruitment is a potential therapeutic target	In DM compared to HC: • CD14+ macrophages were the most abundant cell populations • Followed by myeloid dendritic cells (major producers of IFNß) • And followed by CD14+ CD16+ macrophages. STING production by macrophages has been described as an IFN stimulator. Regarding T cells: predominance of memory T cells (CCR7+ CD45RA-).	histoCAT
	Patel, 2022 (39)	To study similarities and differences between ASyS and DM at the single cell level, by analysing DM-like skin lesions in ASyS patients, and DM lesions.	5 ASyS 7 DM	IMC: Skin biopsies Authors used complementary IF for IFN staining, which showed a similar profile between ASyS and DM.	**In DM vs ASyS:** ↑ myeloid dendritic cell No difference in CD4, CD8 or TREG cells **In ASyS vs DM:** ↑ pSTING+ macrophages with production of IFNß, TNF, IL17	*Standalone IMC analysis*	**Prognosis**: ASYS associated with more fibrosis (and ILD risk) possibly due to the presence of pSTING+ macrophages and IL17 production.	ASyS and DM present a significant overlap, but also some disease specific differences: patients had similar Mph, T cells (CD4, CD8, TREG), B cells and DC infiltration in the skin • DM: increase in myeloid dendritic cell % • Differences in fibrosis and ILD risk between DM and ASyS could be explained by the increased IL17 production in ASyS.	Phenograph
**Myositis and SLE**	Turnier, 2022 (40)	To characterize cell-cell interactions and cellular mechanisms of cutaneous inflammation in DM compared to SLE in children	4 childhood onset CLE 6 JDM	IMC on skin biopsies.	**In child onset CLE**: ↑ CD14+ mph, pDCs, CD8+ cells, B cells **In JDM**: Innate immune signature ↑ CD14+ Mph, ↑CD68+ macrophages Less B cells compared to CLE More endothelial cell - immune cell interactions on skin biopsies;	Complementary microarray.	**Therapeutic:** more disease specific therapeutics in juvenile DM by targeting innate immune cells.	There are immune cell populations differences when comparing CLE and JDM. • JDM had a more prevalent innate immune signature. • CD14+ macrophages are the top immune cell population in JDM; • Interactions between endothelial cells and epithelial cells in DM highlight the skin vasculopathy involved in JDM pathology. • CLE had a higher inflammatory cell infiltrate overall, with increased cell-cell interactions • Microarray confirmed more prominent IFN signature in cSLE compared to DM.	Phenograph
**Myositis**	Throm, 2018 (41)	To assess immune abnormalities in patients affected with JDM	17 treatment-naive JDM 17 HC	CyTOF: PBMC	Signalling differences between JDM and HC involved **PLCγ2 hypophosphorylation** in: • NK cells (majority- • cMo, Mph • CD4+ and CD8+ T cells **In JDM**: ↑Activated NK cells (CD69+, Ki67+)	*Standalone CyTOF analysis*	**Prognosis**: Hypophosphorylation of PLCγ2 was correlated with remission of the disease **Therapeutic**: Possible interest in targeting PLCy2 phosphorylation pathway.	Treatment naive JDM patients present **PLCγ2 hypophosphorylation** which mainly affected NK, and resulted in decreased NK cell cytotoxicity. NK cells were more active in treatment naive JDM (CD69+, Ki67+). Lower levels of NK cells circulation in JDM patients - possibly due to muscle infiltration.	CITRUS, LASSO regression
	Wilfong, 2022 (42)	To identify immune endotypes in IIM through immunophenotyping of PBMCs	17 IIM • 6 DM • 4 PM • 7 ASyS 18 HC	CyTOF: PBMC	**In IIM vs HC:** ↓CD3+ CXCR3+ (including Th1, Th1Th17, and CXC3+ Th2) ↓^†^ decrease in mB cells ↓ RP105/CD180 B cell expression **2 endotypes:** **IIM Endotype 1:** ↑ CXCR4hi across all cellular compartments **IIM Endotype 2:** ↑ DN B cells (CD19+ CD21loCD11c+) ↑ CD3+CD4+PD1+	*Standalone CyTOF analysis*	**Prognosis:** possible link between CXCR4+ expression and severity	Identification of shared immunological features despite different clinical features in IIM. 2 different endotypes of IIM identified, with endotype 2 possibly being pro-fibrotic • Decreased mB cells is a common feature among various types of IIM • Decrease in CXCR3+ T cells: due to possible migration to sites of inflammation	CITRUS, tSNE, marker enrichment modelling
	Maddukuri, 2022 (43)	To investigate the distribution of Lenabasum (CB2R agonist) and response to treatment. Assessment of CB2R expression on various immune cell populations	• 10 DM • 5 HC	IMC: Skin biopsy Complementary IHC and FC for assessment of IFNy IFNß production and confirm CB2R expression.	CB2R expression in DM: Statistically different in all cell populations, but greater in: ↑ in cD123+ pDC and CD11c+ mDC ↑ In B cells	*Standalone CyTOF analysis*	**Therapeutic**: Lenabasum has anti-inflammatory effects shown in vivo and in vitro in DM. Lenabasum downregulates CD4 T cells, and cytokines contributing to DM pathogenesis.	CB2R agonists have anti-inflammatory effects - CB2R expression is increased in DM compared to HC - CB2R expression is higher in the skin compared to the blood of DM patients - and among PBMC: greater expression in dendritic cells compared to lymphocytes (both pDC and mDC) - In the skin (IMC analysis): DC and B cells expressed the most CB2R	Nuclear app-based algorithm for IMC analysis.

ASyS, anti-synthetase syndrome; CB2R, cannabinoid type 2 receptor; CDASI, cutaneous dermatomyositis disease; CITRUS, cluster identification, and characterization and regression; CLE, cutaneous lupus erythematosus; cMo, conventional monocytes; cSLE, childhood-onset systemic lupus erythematosus; DM, dermatomyositis; DN, double negative; HC, healthy controls; IF, immunofluorescence; IFN, interferon; IIM, idiopathic inflammatory myopathy; ILD, interstitial lung disease; IMC, imaging mass cytometry; JDM, juvenile dermatomyositis; LASSO, least absolute shrinkage and selection operator; mB cell, memory B cell; Mph, macrophage; mDC, myeloid dendritic cell; NK, natural killer; PBMC, peripheral blood mononuclear cells; pDC, plasmacytoid dendritic cell; PLCγ2, Phosphatidylinositol-specific phospholipase Cγ2; SLE, systemic lupus erythematosus; STING, stimulators of interferon genes; TNF, tumor necrosis factor; TREG, regulatory T cell.

PBMC analysis performed by Wilfong et al. (42) on blood samples obtained from IIM patients revealed distinct endotypes characterized by different immune cell populations. A particular endotype was rich in DN2 B cells, which were associated with anti-RNP antibody positivity and could hint a pro-fibrotic phenotype.

Systemic treatments in DM, especially in the case of antimalarial resistance, present with numerous side effects. Lenabasum, a non-immunosuppressive cannabinoid type 2 receptor (CB2R) reverse agonist with no psychoactive effect, is currently being investigated as an alternative. IMC has shown a higher CB2R expression in the skin of DM patients, more specifically among DC and B cells, making them a potential target for the drug with sufficient cutaneous tropism (43).

## Systemic vasculitis and inflammatory diseases

**Figure 2:**
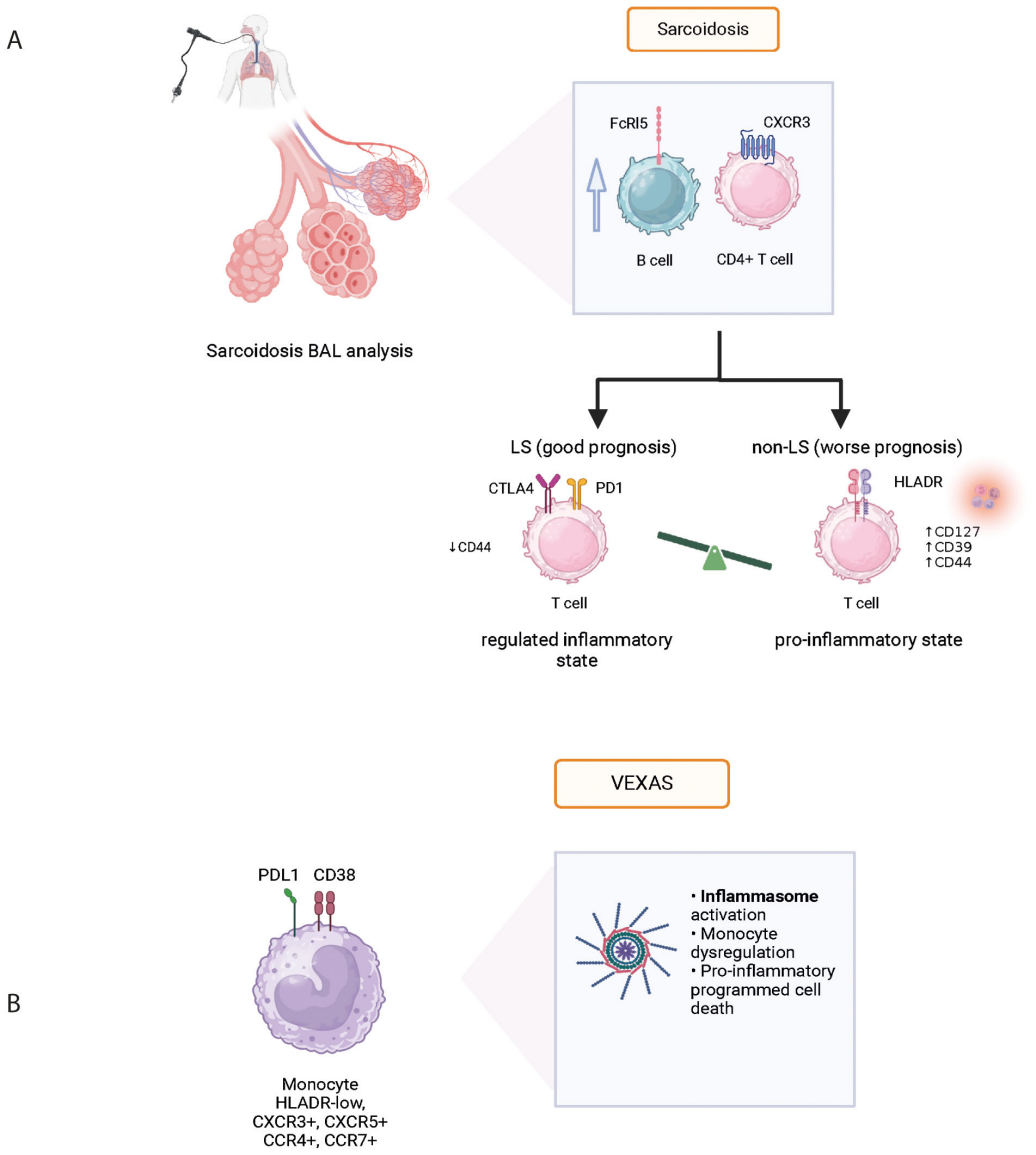
Physiopathological data acquired from mass cytometry in various systemic autoinflammatory diseases: Sarcoidosis **(A)** and VEXAS **(B)**. BAL, bronchoalveolar lavage; FcRl5, Fc Receptor-like 5; CXCR3, C-X-C chemokine receptor 3; CTLA4, cytotoxic T-lymphocyte associated protein 4; VEXAS, vacuoles, E1 enzyme, X-linked, auto-inflammatory, somatic syndrome.

Systemic inflammatory diseases and vasculitis are also associated with sustained and chronic inflammation. Mass cytometry findings have helped identify cellular signature in the blood, bronchoalveolar fluid and tissue analysis (**Table 5** and **Figure 2**).

**Table 5:** Mass cytometry in systemic inflammatory diseases.

Disease	1^st^ author, year	Aim	Number of patients	Technical approach	Abnormalities in cell distribution	Complementary transcriptomic analysis	Impact (prognosis/therapeutic)	Take home message	Algorithm
**Sarcoidosis**	Hata, 2023 (44)	To better understand the immune landscape of ILD satellite of sarcoidosis, in comparison to IPF and ILD linked to CTD using an exploratory mass cytometry analysis	Sarcoidosis: 10 IPF: 8 CTD-ILD: 13 (pSS: 3, DM: 3, SSc: 2, Sharp: 1, SLE: 1, RA: 1) IgG4 RD: 1	CyTOF: whole blood	**In IPF:** ↑* monocytes (CD15hi, CD36hi, CD84hi, CCR2-) ↑ lymphocytes, IL2R+, TIGIT+, LAG3+, CD4+ **In CTD:** ↑ IgG mB cells, ↑ FclR5+ B cells **In 1 SLE-ILD:** ↑ Ratio CD28-/CD4+ **In Sarcoidosis with advanced lesions:** ↑ FcRl5 B cells, and CXCR3+, CD226+, CD4+ T cells	*Standalone CyTOF analysis*	Help with underlying disease diagnosis in case of ILD.	Increase in CXCR3 CD226 CD4 in sarcoidosis • CXCL9 and CXCL11 are ligands of CXCR3, are IFN-inducible and found in granulomas. • CXCR3 expressing CD4+ T cells are recruited in granuloma. B cells: more prevalent in the BAL of sarcoidosis and CTD-ILD patients compared to IPF.	CITRUS
	Kaiser, 2017 (46)	To assess immune phenotype differences in the Löfgren (LS) and non-LS sarcoidosis	4 LS 4 non-LS	CyTOF: BAL	**In 7 clusters more frequent inLSpatients:**↑ CTLA4, PD1, ICOS↓^†^CD44 (adhesion marker)**In 12 clusters found in non-LS patients:**↑ HLADR, CD127, CD39, CD44	*Standalone CyTOF analysis*	Differences in LS and non-LS prognosis could be explained by the pro and anti-inflammatory balance dysregulation.	Identification of 19 individual CD4+ T cell clusters of different abundance between LS and non-LS patients: • LS patients had more expression of immunoregulatory molecules (CTLA4, PD1, ICOS+ TREGS) • Non-LS patients had higher expression of effector markers (HLADR, CD127, CD39, CD44)	CITRUS
**Giant cells arteritis**	Robert, 2022 (47)	Proof of concept for the use of IMC in GCA	[not available]	IMC: 15 marker panel	Identification of 14 distinct clusters of cells in arterial walls, including 8 immune clusters: CD11b+CD66b+ neutrophils, •CD3+CD8+ T cells, •CD3+CD4+ T cells, •CD3+CD4+FOXP3+ TREGS, •CD20+ B cells, •CD3 CD16+ NK cells, •CD11b+CD14+ monocytes •and CD68+ macrophages.	*Standalone IMC analysis*	Ground for future studies using IMC in GCA.	In GCA: Increase in fibroblast and immune cell density (CD4+, activated proliferating T cells, but also myeloid cells and fibroblasts) IMC is an interesting approach to study GCA	Phenograph
**VEXAS syndrome**	Kosmider, 2024 (48)	To explore the inflammatory mechanisms associated with UBA1 somatic mutations and to identify therapeutic targets	40 VEXAS 24 VEXAS-like (autoinflammatory condition without UBA1 mutation) 4 MDS 12 HC	CyTOF: Whole-blood IMC: 3 skin biopsies from VEXAS patients Multiplex cytokine profiling	In VEXAS patients: ↓circulating monocytes ↑ non-classical and exhausted monocytes: ↑ HLADRlow, CD38+ PDL1hi ↑ CXCR3, CXCR5, CCR4, CCR7 ↑ IL1ß and IL18, IL1RA, IL6, IL18	scRNA-seq on monocytes. • ↓ TYROBP/DAP12 • ↓ CTNNB1 • Confirmation of ↑ cellular stress response to unfolded proteins.	Inflammasome pathway targeting in VEXAS Monocyte dysregulation targeting in VEXAS	In VEXAS, circulating conventional monocytes are decreased and dysregulated, with an increase in pro-inflammatory and exhaustion markers. scRNA-seq analysis defective TYROBP/DAP12 and β-catenin signalling pathway and the activation of proinflammatory programmed cell death pathways, as well as cellular stress response to the presence of unfolded proteins.	

BAL, broncho-alveolar fluid; CITRUS, cluster identification, and characterization and regression; CTD, connective tissue disease; CTLA4, Cytotoxic T-lymphocyte associated protein 4; DM, dermatomyositis; FclR5, Fc Receptor-like 5; GCA, giant cell arteritis; HC, healthy controls; HLA, human leukocyte antigen; IFN, interferon; IgG4-RD, IgG4-related disease; ILD, interstitial lung disease; IMC, imaging mass cytometry; IPF, idiopathic pulmonary fibrosis; LS, Löfgren sarcoidosis; mB cells, memory B cells; MDS, myelodysplastic syndrome; NK, natural killer; PD1, programmed cell death protein 1; scRNA-seq, single cell RNA sequencing; TIGIT, T cell receptor immunoreceptor with Ig and ITM domains; SLE, systemic lupus erythematosus; VEXAS, vacuoles, E1 enzyme, X-linked, autoinflammatory somatic syndrome.

### Sarcoidosis

Sarcoidosis is an inflammatory disease of unknown etiology associated with granuloma formation and multi-organ manifestations. Using mass cytometry analysis of immune cell subsets in bronchoalveolar lavage fluid (BALF), the analysis of T and B cells revealed increased levels of CXCR3+ CD226+ CD4+ T cells and the presence of FCRL5+ B cells in sarcoidosis patients with advanced lung lesions (**Figure 2A**). Comparison with idiopathic pulmonary fibrosis (IPF) and connective tissue disease associated interstitial lung disease (ILD) confirmed this activation profile to be unique (44). Interestingly however, despite different histopathological presentations between sarcoidosis and pulmonary tuberculosis, authors found no significantly different gene expression profile when performing spatial transcriptomic analysis of the lung granuloma from patients presenting these conditions (45). Although pulmonary manifestations are very common in sarcoidosis and often contribute to comorbidity, disease phenotype can differ greatly between individuals. Löfgren syndrome (LS) stands out with a notably favorable prognosis and distinct clinical presentation including erythema nodosum and/or ankle arthritis. In their study, Kaiser et al. (46) performed mass cytometry on BALF which revealed a higher expression of anti-inflammatory molecules CTLA-4, PD1, and ICOS-mediated TREG activation in LS compared to other forms of sarcoidosis (**Table 5**). This finding provides insights into the varying prognosis observed between LS and non-LS forms of sarcoidosis.

### Large vessel vasculitis (LVV)

Using highly multiplexed imaging mass cytometry, a comprehensive analysis of cell lineages involved in giant cell arteritis (GCA) pathogenesis has been performed (47). They show an *in-situ* signature in GCA identified by unbiased cell clustering (**Table 5**). A hierarchical clustering based on cell type abundance analysis identified two distinct cluster communities in GCA patients: one made of lymphocytes, neutrophils and endothelial cells, and one made of myeloid cells, vascular smooth muscle cells, pericytes, fibroblasts and myofibroblasts. A link with clinical presentation and therapeutic response has yet to be established.

### VEXAS syndrome

VEXAS (Vacuole, E1 enzyme, X-linked, autoinflammatory, somatic) syndrome has been associated with various clinical manifestations including fever, neutrophilic cutaneous lesions, arthralgias, pulmonary inflammation, chondritis, and vasculitis. These manifestations are related to the recruitment and accumulation of inflammatory and innate immune cells in targeted tissues. In a recent prospective cohort of VEXAS patients, Kosmider et al. used an integrative approach based on clinical and biological data, in-depth phenotypical analysis of whole blood immune cells, cytokine profiling, single-cell RNA sequencing, and tissue imaging by mass cytometry on skin biopsies, to explore the inflammatory mechanisms associated with *UBA1* somatic mutations in VEXAS patients and to identify therapeutic targets (48). Dimensionality reduction analysis revealed significant differences in the repartition of monocyte subsets in VEXAS patients with a decrease in circulating monocytes, primarily involving intermediate (CD14+ CD16+) and nonclassical (CD14^lo^ CD16+) monocytes, but with high expression of CXCR3, CXCR5, CCR4 and CCR7 (**Figure 2B**). Using an integrative approach with complementary scRNA-seq, the authors were able to identify molecular pathways involved in monocyte dysregulation at the single cell level including TYROBP/DAP12, a pathway involved in myeloid cell function, and CTNNB1, involved in cellular homeostasis. In addition, the authors used a combination of markers in IMC to determine the anatomical localization of inflammatory cells within target tissues from VEXAS patients. They demonstrated that CD16+ CD163+ monocytes and proinflammatory M1 macrophages were abundantly present in skin lesions, expressing granzyme B which could be associated with pathogenicity in macrophage functions (**Table 5**). These results demonstrate that *UBA1*-mutated inflammatory monocytes aberrantly expressing chemokine receptors could be attracted into target tissues and promote local inflammation, contributing to the clinical presentation.

## Discussion

Auto-immune and inflammatory diseases are extremely complex in nature and involve a high number of actors of the immune system. Most of the CyTOF analyses mentioned in this review were exploratory in nature, derived from studies conducted on highly selected populations with limited sample sizes. Conventional flow cytometry, despite its limitations, has provided great pathophysiological insights regarding the mentioned pathologies. However, the high dimensionality of mass cytometry provides better coverage of immune population dynamics, with sufficient power to identify rare cell types compared to flow cytometry. Though the results of the studies mentioned throughout this review are purely descriptive and have yet to translate into real-life practice, CyTOF has the power to help to identify therapeutic targets, as well as cell populations associated with refractory status as well as prognostic factors. In combination with other high throughput -omic strategies, CyTOF has the potential to help stratify future trials with the administration of targeted treatments based on immune cell phenotype. Early discrimination of patients likely to respond well to a given therapeutic option from patients unlikely to benefit, could contribute to more personalized and tailored therapeutic schemes.

### CyTOF characteristics

CyTOF is a recently introduced high-dimensional technology which enables the use of a high number of markers, due to the absence of overlap and autofluorescence. The volume of data generated by the analysis of marker expression in millions of cells can increase significantly; however, within the realm of mass cytometry, the array of markers remains finite, typically capped at a maximum of 50. While data analysis is based on an unsupervised approach, there is always a need for scientists to develop a CyTOF panel based on their initial hypotheses on which cellular actors are implicated in the different physiological processes. Such hypotheses often stem from previously reported literature based on lower dimensional technologies, such as conventional flow cytometry. CyTOF analysis, even when performed on small cohorts, generates a high amount of data, with interpretation requiring strong bioinformatics expertise. Required skills include data cleaning, normalization, dimensionality reduction, and statistical analysis as well as means to ensure data integrity and security. To handle such wealth of generated data with a robust workflow, a multidisciplinary team involving clinicians, biologists, and biostatisticians is often required. Moreover, available algorithms must be employed with care and distance, favoring the most appropriate modeling strategy associated with the least bias considering the experimental design.

CyTOF findings sometimes require confirmation using lower dimensional techniques such as flow cytometry on validation cohorts. CyTOF differs from conventional flow cytometry, mainly due to its destructive nature: after nebulization and cell atomization, cells cannot be sorted for further analysis, thus limiting their use for direct complementary explorations. There is a great interest however, in combining CyTOF with other omics technologies in the context of autoimmune diseases, in a multiscale approach to cell biology entitled “Systems Immunology” with great granularity.

CyTOF analysis can be performed on whole blood samples, PBMCs, biological fluids (including saliva, bronchial alveolar fluid, and urine), suspension of cells obtained from tissues, and whole tissue biopsies. Performing CyTOF on non-blood samples presents with a few challenges, including the smaller number of cells available due to a lower concentration and a lower sample volume, as well as the varying difficulty to access such samples. Moreover, the analysis requires fluid- or tissue-specific protocols designed to ensure the elimination of cellular debris (often abundant in broncho-alveolar mucus for instance), while ensuring the preservation of rare cell populations.

### CyTOF and single-cell transcriptomics

Mass cytometry has emerged as a key tool to profile multiple parameters of the immune system and allows the identification, quantification, and phenotypical characterization of a wide range of immune cell subsets simultaneously. Mass cytometry also has the potential to help assess cellular response to immunotherapy in the context of autoimmune diseases. B-cell depleting therapies have been extensively used and studied in the context of systemic diseases, however several patients do not respond well and drug-free remission is rarely observed residual disease is frequent. More recently, CAR-T cells have been studied in refractory SLE patients. CyTOF studies in the context of this novel treatment have the potential assess the cytotoxicity and cytokine release following CAR-T cell therapy.

**Figure 3:**
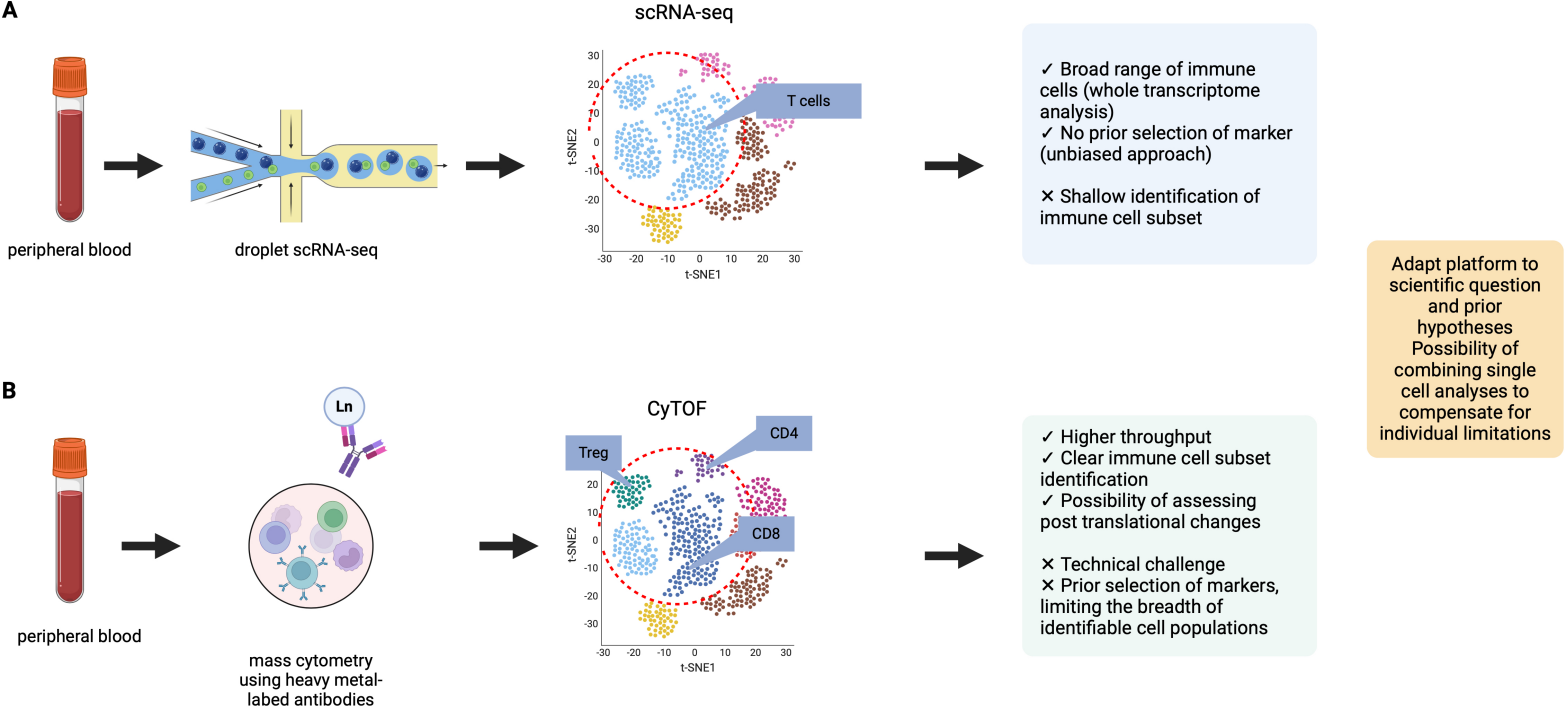
Comparison of two single-cell analysis modalities: CyTOF and scRNA-seq. Data were adapted from *Kashima* et al. (49) **(A)** Representation of data obtained from droplet scRNA-seq. scRNA-seq allows for the analysis of a broad range of immune cell populations, in an unbiased approach with no required panel design. **(B)** Representation of data obtained from mass cytometry analysis. Mass cytometry can perform analysis at the protein level, with clear immune cell population identification (for example: clear definition of T cell subsets). In the example provided, the t-SNE representation of CyTOF data shows more heterogeneity in cell cluster identification. Advantages and limitations of both single-cell approaches are presented.

Other -omic strategies, including genomics and transcriptomics, have led to a better understanding of the genotype and phenotype of cells. The primary difference compared to CyTOF is the lack of accounting for post-translational changes in proteins at the cellular level, including phosphorylation, which are often a better representation of changes in the context of biological function. Proteins are also often the therapeutic targets of treatment. ScRNA-seq, a technique not restricted to a 40-markers predetermined panel, represents a more unbiased approach thus allowing for a broad range of cellular populations. The two single cell approaches were directly compared in a study performed to study the tumor microenvironment of gastric cancer (49). In this study, CyTOF displayed better performance for clear identification of distinct cell subsets whereas scRNA-seq detected cellular populations not included in the CyTOF panel. The complementarity of these two approaches had potential to better assess the tumor microenvironment of gastric cancer. In another study, performed on synovial cells obtained from rheumatoid arthritis patients, the authors first performed a scRNA-seq analysis on previously sorted fibroblasts, monocytes, B and T cells, leading to the identification of 16 scRNA-seq clusters (50). In a unified approach, they then performed CyTOF which identified 32 clusters, all related to the previously described scRNA-seq clusters, indicative of additional heterogeneity, better assessed with protein levels expression. Additionally, when stratifying patients depending on the level of synovial leukocyte infiltration (leukocyte-rich vs. leukocyte-poor), the significantly differentially abundant clusters were the ones assessed using CyTOF and thus linked to protein expression. We represent the respective limitations and advantages of single cell RNAseq and CyTOF in **Figure 3**. While scRNA-seq enables the assessment of thousands of genes with the possibility of performing whole transcriptome analysis, it is at the cost of sparse data. Mass cytometry measures the expression of 40 pre-selected markers on hundreds of thousands of cells, an approach which does not allow the identification of cell populations not included within the panel.

Combining single-cell data obtained at the transcriptome level, and cellular protein expression level using mass cytometry has the potential to help overcome limitations linked to each individual approach. A systems approach to immunological biology, combining the multiscale study of the immune phenotypes in various diseases using multiple -omic technologies while addressing their respective limitations, could enhance our understanding of biological processes in the context of autoimmune and autoinflammatory systemic diseases.

### Single-cell spatial multi-omics

Comparative tissue analysis using imaging mass cytometry are a valuable tool for gaining a better understanding of the physiopathology of auto-immune diseases, including immune cell infiltration and tissue-specific signaling. The existence of a gradient between blood and tissue infiltration of immune cells can be an exploratory evidence of cell migration. Such findings can be validated using more widely available techniques such as immunohistochemistry on a selected number of markers.

Spatial transcriptomics, in contrast, enables transcriptome-wide profiling from tissue sections. While resolution recent technological advances have significantly improved spatial resolution (51), most platforms remain at a “supra-cellular” resolution ranging from 40 – 100 µm (52), compared to the subcellular resolution of IMC (53). IMC provides an understanding of the heterogeneity of protein expression among different cell subsets, with the ability to study post-translational and -transcriptional modifications under specific pathological conditions while retaining spatial information. This makes IMC invaluable for functional protein analyses using pre-established antibody panels. IMC has helped to contribute uniquely to the understanding of immune disease, especially to establish the impact of spatial interactions with the possibility of studying in detail target-organs. In the context of CLE, *Patel* et al. were able to assess cell-cell interactions and avoidances using neighborhood analysis (35). The presence of these cellular interactions had direct clinical impact, as within patients who did not respond to hydroxychloroquine, γ∂ T cells did not interact with CD8+ T cells, interacted less with CD4+ T cells and cDCs, but displayed an increased interaction with CD14+CD16+ macrophages and endothelium, indicating infiltration.

Spatial transcriptomics provides significantly broader information due to its ability to generate genome-wide transcriptomic data. This unbiased approach is unrestricted by pre-defined ~40-antigen panels, making it well-suited for exploratory analyses and the identification of novel biomarkers. In a recent IMC study performed the skin lesions of SSc patients, authors identified additional subpopulations of fibroblasts with altered proportion in SSc skin compared to previous reports in the literature, including Thy1+; ADAM12^high^; PU.1^high^ fibroblasts, ADAM12+;GLI1+ fibroblasts and TFAM^high^ fibroblasts (54). While these specific fibroblast subsets were previously unreported, a previous spatial transcriptomic study had revealed the compartmentalization of fibrotic processes in the skin of SSc patients, as well as the underlying involvement of the Hippo gene pathway in pro-fibrotic myofibroblast differentiation, and endothelial to mesenchymal differentiation (55).

While IMC requires time-consuming data acquisition and analyzes fewer cells per sample, spatial transcriptomics processes larger areas of tissue and profiles a higher number of cells per section. Ultimately, these complementary technologies cater to distinct research goals: IMC for detailed protein phenotypic studies and spatial transcriptomics for high-throughput transcriptomic exploration. IMC and spatial transcriptomics can work synergistically to provide complimentary insights on cell phenotype at the protein and the mRNA level respectively.

## Conclusion

The identification of biomarkers able to stratify patients into clinically relevant groups is a major research interest in autoimmune and inflammatory diseases. Future studies could include mass cytometry findings, combined to other omic strategies, to stratify patients in therapeutic trials to provide advancements in personalized therapies in the field of auto-immune and inflammatory diseases. Moreover, CyTOF brings the opportunity to explore refractory status in systemic diseases, thus offering the opportunity for precision medicine. One obstacle limiting the widespread adoption of CyTOF is its high associated cost, though decreasing time and expense needed to generate datasets have helped create both exciting opportunities and great challenges for scientists.
